# Translation efficiency of heterologous proteins is significantly affected by the genetic context of RBS sequences in engineered cyanobacterium *Synechocystis* sp. PCC 6803

**DOI:** 10.1186/s12934-018-0882-2

**Published:** 2018-03-02

**Authors:** Kati Thiel, Edita Mulaku, Hariharan Dandapani, Csaba Nagy, Eva-Mari Aro, Pauli Kallio

**Affiliations:** Molecular Plant Biology, Department of Biochemistry, University of Turku, 20014 Turku, Finland

**Keywords:** Ribosome binding site, Translational efficiency, Genetic context, *Synechocystis* sp. PCC 6803

## Abstract

**Background:**

Photosynthetic cyanobacteria have been studied as potential host organisms for direct solar-driven production of different carbon-based chemicals from CO_2_ and water, as part of the development of sustainable future biotechnological applications. The engineering approaches, however, are still limited by the lack of comprehensive information on most optimal expression strategies and validated species-specific genetic elements which are essential for increasing the intricacy, predictability and efficiency of the systems. This study focused on the systematic evaluation of the key translational control elements, ribosome binding sites (RBS), in the cyanobacterial host *Synechocystis* sp. PCC 6803, with the objective of expanding the palette of tools for more rigorous engineering approaches.

**Results:**

An expression system was established for the comparison of 13 selected RBS sequences in *Synechocystis*, using several alternative reporter proteins (sYFP2, codon-optimized GFPmut3 and ethylene forming enzyme) as quantitative indicators of the relative translation efficiencies. The set-up was shown to yield highly reproducible expression patterns in independent analytical series with low variation between biological replicates, thus allowing statistical comparison of the activities of the different RBSs in vivo. While the RBSs covered a relatively broad overall expression level range, the downstream gene sequence was demonstrated in a rigorous manner to have a clear impact on the resulting translational profiles. This was expected to reflect interfering sequence-specific mRNA-level interaction between the RBS and the coding region, yet correlation between potential secondary structure formation and observed translation levels could not be resolved with existing in silico prediction tools.

**Conclusions:**

The study expands our current understanding on the potential and limitations associated with the regulation of protein expression at translational level in engineered cyanobacteria. The acquired information can be used for selecting appropriate RBSs for optimizing over-expression constructs or multicistronic pathways in *Synechocystis*, while underlining the complications in predicting the activity due to gene-specific interactions which may reduce the translational efficiency for a given RBS-gene combination. Ultimately, the findings emphasize the need for additional characterized insulator sequence elements to decouple the interaction between the RBS and the coding region for future engineering approaches.

**Electronic supplementary material:**

The online version of this article (10.1186/s12934-018-0882-2) contains supplementary material, which is available to authorized users.

## Background

In response to increasing environmental concerns and exponentially growing demand for consumer products, there is an urgent global need to find sustainable alternatives for different carbon-based chemicals which are currently derived from non-renewable sources. As part of this development, photosynthetic cyanobacteria have been studied as potential next-generation biotechnological host organisms for the production of desired chemicals directly from atmospheric CO_2_ and water, using solar radiation as energy [1, 2]. Although the proof of concept has been established for the technology, there are still various biological and technical shortcomings which critically restrict us from harnessing the photosynthetic capacity to a sufficient degree for commercial applications. In particular, the efficiency of converting the light energy into the target products is currently inadequate, and further development calls for more flexible strategies to enhance preparative throughput and expand chemical diversity beyond the conventional engineering approaches in cyanobacterial research. In order to overcome the constraints, systematic synthetic biology approaches relying on validated genetic control elements, modular assembly systems and optimized expression strategies are currently being evaluated in cyanobacterial hosts such as *Synechocystis* sp. PCC 6803 (*Synechocystis* from here on).

One of the typical challenges in metabolic engineering is to have precise control over the expression of the introduced genes in a predictable manner. Besides promoters which typically serve as the master switches in regulating expression at the transcription phase, *ribosome binding site* (RBS) sequences play a key role in determining the output by controlling the translation efficiency of individual ORFs. In addition to maximizing the expression level of a specific target protein, RBSs can be used for modulating the relative translation efficiencies of individual proteins in polycistronic pathways at a broad dynamic range [3]. Importantly, this provides the means for optimizing the performance of engineered pathways with multiple heterologous genes, in which the metabolic flux through the subsequent enzyme-catalyzed biosynthetic steps need to be balanced in order to function properly in context with the surrounding cellular metabolism [3]. However, unlike for established model organisms such as *E. coli* for which structure–function relationships in genetic regulation have already been extensively studied, the corresponding information on the function of RBSs in cyanobacteria is still rather limited to allow complex rational engineering.

Several studies have previously addressed the function and relative activities of RBSs in *Synechocystis* (Table 1) [4–7], but in many cases the RBSs and promoter sequences are either discussed together without clear distinction, or direct assessment of factors affecting the translational efficiency is difficult due to variation in context or experimental set-up between the publications. Although the nucleotide sequences around the RBS region are known to potentially impact the level of translation [6, 8, 9], the phenomenon has not been systematically evaluated in cyanobacteria, and there is no clear consensus of the frequency or extent of the effect in engineered cyanobacterial systems. With the realization that the function of RBSs can be clearly species-specific, as observed for example between *E. coli* and *Synechocystis* [4, 10, 11], such information could help to identify the most critical points in the design of new context-independent expression strategies. Currently, even though prominent in silico prediction tools have been developed and optimized to evaluate the function of RBS sequences [12–14], the correlation with the observed activities in cyanobacteria appear to be poor [15, 16], and should be used with caution if direct experimental validation is not available.

**Table 1:** RBSs subjected to in vivo characterization or comparative analysis in *Synechocystis* sp. PCC 6803 as reported in literature

Name and sequence	Organism/source	Reporter	References
BBa_B0029 ATTCACACAGGAAACCTACTAGATG NTTCACACAGGAAACCTCTAGAATG	BioBrick	mTagBFP EYFP	[6] [6]
BBa_B0030 (N)_n_ATTAAAGAGGAGAAA(N)_n_**ATG** AATTAAAGAGGAGAAATACTAG**ATG** NATTAAAGAGGAGAAATCTAGA**ATG**	BioBrick	GFPmut3b mTagBFP EYFP	[4] [6] [6]
BBa_B0031 GATCACACAGGAAACCTACTAG**ATG** NNTCACACAGGAAACCTCTAGA**ATG**	BioBrick	mTagBFP EYFP	[6] [6]
BBa_B0032 (N)_n_TCACACAGGAAAG(N)_n_**ATG** AGATCACACAGGAAAGTACTAG**ATG** NNNTCACACAGGAAAGTCTAGA**ATG**	BioBrick	GFPmut3b mTagBFP EYFP	[4] [6] [6]
BBa_B0033 CTAGATCACACAGGACTACTAG**ATG** NNNNNTCACACAGGACTCTAGA**ATG**	BioBrick	mTagBFP EYFP	[6] [6]
BBa_B0034 (N)_n_ AAAGAGGAGAAA(N)_n_**ATG** TAGAAAAGAGGAGAAATACTAG**ATG** NNNNAAAGAGGAGAAATCTAGA**ATG**	BioBrick	GFPmut3b mTagBFP EYFP	[4] [6] [6]
BBa_B0035 GAATTAAAGAGGAGAATACTAG**ATG** NNATTAAAGAGGAGAATCTAGA**ATG**	BioBrick	mTagBFP EYFP	[6] [6]
BBa_B0064 TAGAAAAGAGGGGAAATACTAG**ATG** NNNNAAAGAGGGGAAATCTAGA**ATG**	BioBrick	mTagBFP EYFP	[6] [6]
*psbA2* RBS AGACATAAGGAATTATTACTAG**ATG** NNNCATAAGGAATTATTCTAGA**ATG**	*psbA2* ^a^	mTagBFP EYFP	[6] [6]
*rbcL* RBS AGATTATGGAGGACTGTACTAG**ATG** NNNTTATGGAGGACTGTCTAGA**ATG**	*rbcL* ^a^	mTagBFP EYFP	[6] [6]
RBS* NNNNNNTAGTGGAGGTNNNNNN**ATG** TCTAGATAGTGGAGGTTACTAG**ATG** NNNNNNTAGTGGAGGTTCTAGA**ATG**	A synthetic RBS^a^	GFPmut3b mTagBFP EYFP	[4] [6] [6]
Unnamed NA*/*GAAGGAGGTNNNNNNNNNNNN**ATG**	A synthetic RBS	eCFP and YFP	[5]
RBSv4 AAAATAAAGAAGGAGGAACAGC**ATG**	A synthetic RBS	Synthetic *efe*	[7]

The sequences (22 bp upstream from the initiation codon) are presented in 5′–3′ direction, with the core SD consensus sequence GGAGG underlined and ATG shown in bold. The nucleotides that have not been specified in the corresponding publications are denoted as N. Comparative studies in which the RBS is not clearly distinguished from the context of the promoter have not been included

^a^Indicates that the corresponding gene has been used also as the source of RBS sequence in this study

RBS is a short (typically less than 20 bp) nucleotide sequence at the 5′ UTR of mRNA, which guides the ribosome binding in the correct orientation in respect to the start codon, and thus allows translation to begin. As the initiation phase is the key rate-limiting step in translation, RBS constitutes an important control element in determining the accuracy and overall efficiency in protein expression. RBSs typically contain a specific guanidine-rich core region called Shine-Dalgarno (SD) sequence, located about 5–13 bp upstream from the initiation codon. The consensus sequence for SD is 5′-GGAGG-3′ [4, 11, 17] which is recognized and bound by the complementary 3′ anti-SD sequence of the 16S rRNA of the 30S ribosomal subunit, resulting in the translation initiation complex formation. The efficacy of the primary interaction between the RBS and the ribosome depends on the (i) degree of complementarity between the SD core sequence and the 16S rRNA, (ii) the distance of the SD from the translation initiation codon, and (iii) the surrounding nucleotide sequences, which may form secondary structures that interfere with the binding [4, 11, 17]. Despite high level of conservation in the SD sequences between prokaryotes, most RBSs show variations in the region. For example, while *E. coli* and *Synechocystis* share the same core anti-SD sequence corresponding to 5′-GGAGG-3′, the respective percentage of genes carrying this sequence in the two organisms is 57 and 26%, respectively. In addition, the optimal aligned spacing between the start codon and the SD core sequence has been reported to be 7–9 bp for *E. coli* but 9–11 bp for *Synechocystis* [11].

The objective of this study was to expand the current knowledge on the factors influencing translational efficiency in the cyanobacterium *Synechocystis.* This was to be accomplished by systematic quantitative comparison of a set of selected RBS sequences in vivo in order to (i) find specific sequences allowing the highest translational efficiencies, (ii) define a series of RBSs covering a dynamic range of different expression levels, and (iii) evaluate the extent to which the nucleotide sequence of the target gene affects the outcome. The aim was to compile a library of alternative expression constructs harboring an array of RBSs fused with fluorescent reporter genes sYFP2 and GFPmut3b (YFP and GFP from here on, respectively) as quantitative indicators of translation efficiency, followed by generation and fluorometric characterization of the corresponding *Synechocystis* strains. The experimental study was to be complemented by comparative bioinformatic analysis to correlate between the RBS sequences and obtained expression levels, and to predict possible secondary structures between the coding sequence and the upstream region to explain observed differences.

## Results

### Selecting RBSs for in vivo comparison in *Synechocystis*

Thirteen different RBS sequences were selected for the comparative study to determine quantitative differences in translation efficiencies in *Synechocystis* (Table 2). Six of the sequences form a degenerative series that has previously been evaluated in *E. coli*, where they span an expression level range of several magnitudes (A–E, Z; in decreasing order of efficiency) [3, 12]. The remaining seven RBS sequences derived either directly from native highly expressed cyanobacterial genes (Table 2; S2–S5), or from expression constructs designed for *Synechocystis* (Table 2; S1, S6, S7), and had not previously been systematically compared for efficiency. The RBS sequences were composed of 12–22 bp variable region immediately upstream from the start codon ATG (Table 2), preceded by an invariant 5′ upstream spacer-insulator region [3]. The sequences were ordered as synthetic DNA fragments, flanked by specific restriction sites, which could be directly used for the generation of the expression construct library.

**Table 2:** The RBS sequences compared in this study

Name and sequence	Gene/source	Genebank ID/references
S1 CTAGAGTAGTGGAGGTTACTAG**ATG**	Synthetic RBS based on *Synechocystis* anti-SD	[4, 25]
S2 AATATAAGTAGGAGATAAAAAC**ATG**	*cpcB* from *Synechococcus* sp. PCC 7002	CP000951.1
S3 AGTCAAGTAGGAGATTAATTCA**ATG**	*sll1577, cpcB* from *Synechocystis*	U34930.1
S4 ATACATAAGGAATTATAACCAA**ATG**	*slr1131, psbA2* from *Synechocystis*	X13547.1
S5 TGTTTATGGAGGACTGACCTAG**ATG**	slr0090, *rbcL* from *Synechocystis*	X65960.1
S6 TAGCCTAGGAGGAGGAAAAATC**ATG**	Synthetic RBS	Unpublished construct
S7 CATTAAAGAGGAGAAAGGTACC**ATG**	pDF-lac	[20]
A AACAAAATGAGGAGGTACTGAG**ATG**	Adapted from Salis et al. [12]	[3]
B *TTTAACTTTA*AGGAGGTTTGGA**ATG**	Adapted from Salis et al. [12]	[3]
C *ACTTTA*AAGTTAAGAGGCAAGA**ATG**	Adapted from Salis et al. [12]	[3]
D *TTTA*TAAGCAGGACCGGCGGCG**ATG**	Adapted from Salis et al. [12]	[3]
E *TAACTTTA*TTCGCAGGGGGAAG**ATG**	Adapted from Salis et al. [12]	[3]
Z *TTTAACTTTA*CACCATACACTG**ATG**	Adapted from Salis et al. [12]	[3]

S2–S5 originate directly from the native cyanobacterial genes and correspond to the NCBI Genbank sequences (22 bp preceding the start codon), while S1 and S6–S7 derive from expression constructs designed earlier for *Synechocystis* sp. PCC 6803. RBS A–E and Z represent sequences which have earlier been characterized on *E. coli*. The core SD consensus sequence GGAGG has been underlined, start codon ATG shown in bold, and the upstream insulator sequence identical in all constructs shown in italics

### Generation of the expression strains for evaluating the RBS efficiencies

In order to evaluate the efficiency of the designed RBSs in *Synechocystis* in vivo, each of the sequences was first fused with a gene coding for the fluorescent reporter protein YFP [18] and a codon-optimized counterpart GFP (Additional file 1) [19]. These two reporters were specifically designed to have low nucleotide sequence similarity between one another to study the influence of the genetic context (i.e. the combined effect of the RBSs with the coding sequence) on the outcome. To enable expression in *Synechocystis*, the fragments were transferred into a pDF-lac [20]—derived expression plasmid pDF-lac2, under the control of the IPTG inducible Lac promoter variant P_A1lacO-1_. The generated 26 plasmid constructs (Additional file 2) were then transformed into *Synechocystis*, and the resulting antibiotic resistant strains were verified by colony PCR (Additional file 3: A, B).

### Analytical set-up for the comparative analysis of RBSs

To confirm the function of the expression systems and to optimize the analytical set-up, the 26 generated *Synechocystis* strains were subjected to a series of fluorescence spectroscopy measurements. Time course analysis between 0 and 6 h after induction (Additional file 4) showed a clear fluorescence response for majority of the strains in respect to YFP and GFP measured at 495 nm (ex.)/535 nm (em.) and 485 nm (ex.)/525 nm (em.), respectively. Based on the response profiles, the 6-h mark was selected as the default time-point for the quantitative comparison of the strains in successive experiments, due to sufficiently broad intensity range and signal levels which remained clearly below the saturation limit. Comparison of the induced and uninduced fluorescent profiles showed that the promoter P_A1lacO-1_ was adequately repressed under the experimental conditions (Additional file 4; see dotted lines), confirming that unspecific background fluorescence did not have any apparent effect on the data interpretation. In addition, the differences in growth of the parallel strains appeared to be insignificant and without direct correlation with the observed expression levels (Additional file 5), yet to ensure the validity of the comparison, the fluorescence signals were in each case normalized to OD _750 nm_.

### System validation by two parallel analytical approaches

**Fig. 1:**
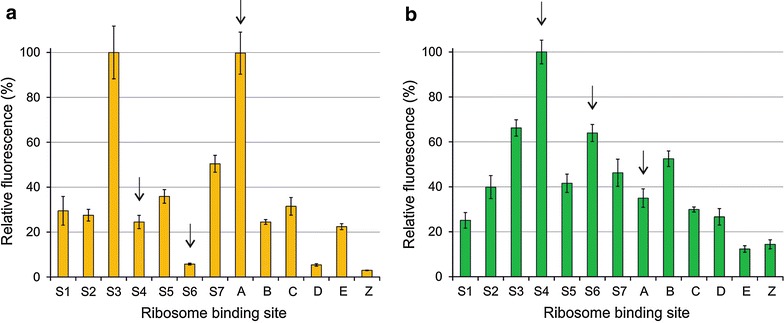
Relative fluorescence (*full dataset*) representing the translational efficiencies of the 13 RBSs in engineered *Synechocystis* sp. PCC 6803 strains. Two alternative fluorescence reporters used in the study were **a** sYFP2 and **b** GFPmut3b, measured from intact cells at 6 h after induction. The bars showing the average relative fluorescence values (%) and standard deviations were calculated from six parallel biological replicates and three technical replicates (n = 18) normalized to OD_750 nm_. The arrows indicate the RBSs which differ the most between the two datasets

**Fig. 2:**
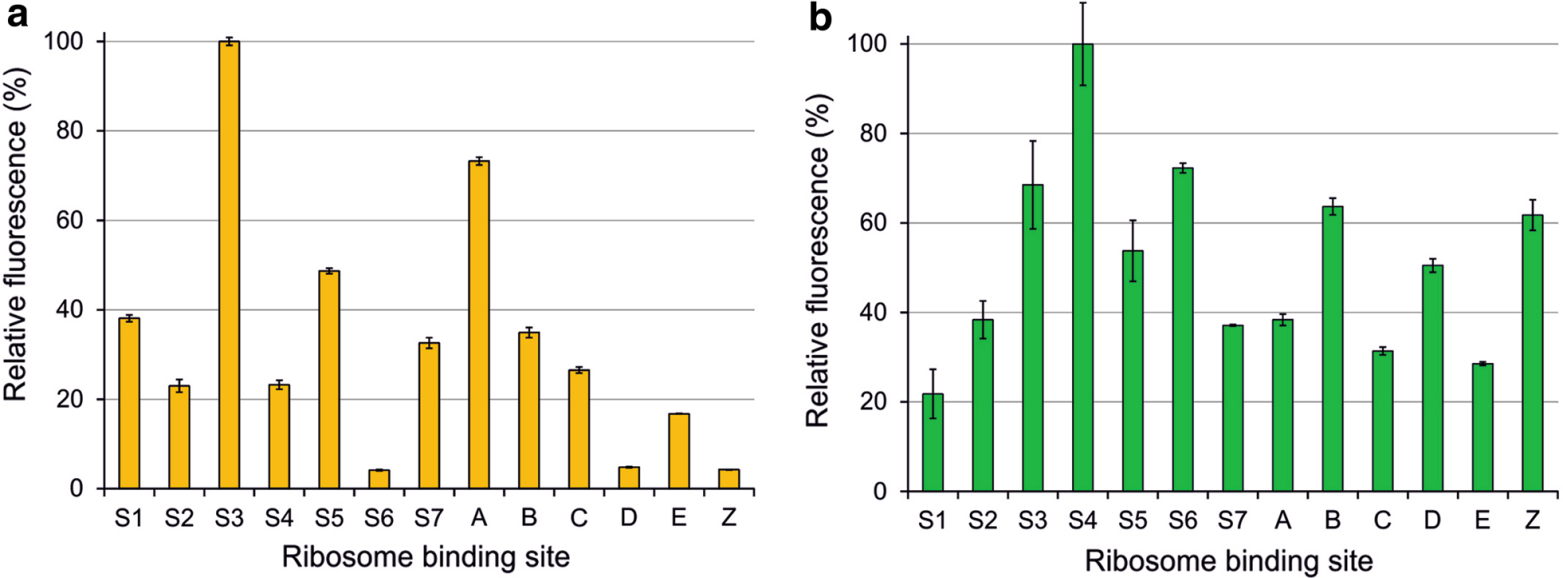
Relative fluorescence (*one*-*day dataset*) representing translational efficiencies of the 13 RBSs in engineered *Synechocystis* sp. PCC 6803 strains. Two alternative fluorescence reporters used in the study were **a** sYFP2 and **b** GFPmut3b, measured from intact cells at 6 h after induction. The bars showing the average relative fluorescence values (%) and standard deviations were calculated from one biological replicate and three technical replicates (n = 3) and normalized to OD_750 nm_

The 26 generated *Synechocystis* strains with 13 distinct RBS sequences regulating the translation of YFP and GFP were subjected to two independent rounds of quantitative analysis in order to confirm the validity of the expression-level comparison. In the first approach (i.e. *full dataset*), each of the 13 strains was characterized individually on separate days, using six biological replicates with three technical replicates (n = 18), for both YFP (Fig. 1a) and GFP (Fig. 1b). In the second approach (i.e. *one*-*day dataset*) all the 13 strains were analyzed in parallel on the same day but with only three technical replicates (n = 3) for YFP (Fig. 2a) and GFP (Fig. 2b). The primary observation was that, besides the broad range of expression levels, the variation between the biological replicates within each full dataset was negligible (Fig. 1 and Additional file 4). This indicated low clonal variation and high reproducibility, which were essential for meaningful statistical evaluation of the RBSs. Comparison of each full dataset (Fig. 1) with the corresponding one-day dataset (Fig. 2), on the other hand, showed that the RBS-specific expression profiles were highly similar between the two approaches even by mere visual evaluation. Statistical analysis conducted by two independent methods further verified high correlation between the full dataset and the one-day dataset with high confidence (p values under 0.001) (Table 3). This demonstrated that the two alternative experimental approaches resulted in a very similar outcome, and individually represented the relative RBS-specific expression trends to a reliable degree. For maximal statistical significance, the full datasets with six biological replicates (Fig. 1) were used for the subsequent rounds of analysis.

**Table 3:** Statistical comparison between the *full dataset* and the *one-day dataset* of sYFP2 and GFPmut3b fluorescence profiles using Pearson correlation coefficient and Spearman’s rank correlation coefficient

	YFP: full dataset vs 1-day dataset		GFP: full dataset vs 1-day dataset	
Method	correlation	p-values	correlation	p-values
Pearson	0.942	< 0.0001	0.801	0.001
Spearman	0.915	0.0002	0.918	< 0.0001

### The YFP and GFP expression profiles are clearly distinct

The YFP and GFP genes used in this study did not share any significant nucleotide sequence similarity due to GFP codon optimization, and consequently, could be used as representatives of unrelated target genes to study the effect of the downstream coding region on the RBS-specific expression. Comparison between the YFP (Fig. 1a) and GFP fluorescence datasets (Fig. 1b) revealed distinct divergence in the overall shapes of the profiles, reflecting pronounced gene-specific differences in the expression of different RBS strains. As further verified by the low statistical correlation between the YFP and GFP datasets (Table 4), the expression levels were clearly not determined by the specific RBS sequences alone, but also significantly affected by the downstream gene sequence. At the level of individual strains, while the relative performance of many of the 13 RBSs appeared to be similar with YFP and GFP (Fig. 1), the translational efficiency recorded for several specific constructs was dramatically affected by the downstream region. The most distinct differences were observed for RBSs S4, S6 and A (Fig. 1; see arrows) which performed almost in an opposite manner in respect to the expression of YFP and GFP. Notably, exclusion of these RBSs from the comparison (Table 4) markedly improved the statistical correlation and p-values, reflecting the degree of underlying similarity between the YFP and GFP profiles, and ultimately in the efficiency of individual RBSs which may be easily masked by context-dependent effects.

**Table 4:** Statistical comparison between the sYFP2 and GFPmut3b fluorescence profiles (*full dataset*) using Pearson correlation coefficient and Spearman’s rank correlation coefficient

Method	Full dataset		Full dataset excluding S4 + S6 + A	
	YFP vs GFP		YFP vs GFP	
	Correlation	p value	Correlation	p value
Pearson	0.186	0.346	0.781	0.008
Spearman	0.543	0.243	0.697	0.029

The table shows the values calculated based on the entire dataset, and after the exclusion of the most divergent RBSs (S4, S6 and A)

### Confirmation of the functional context dependence

**Fig. 3:**
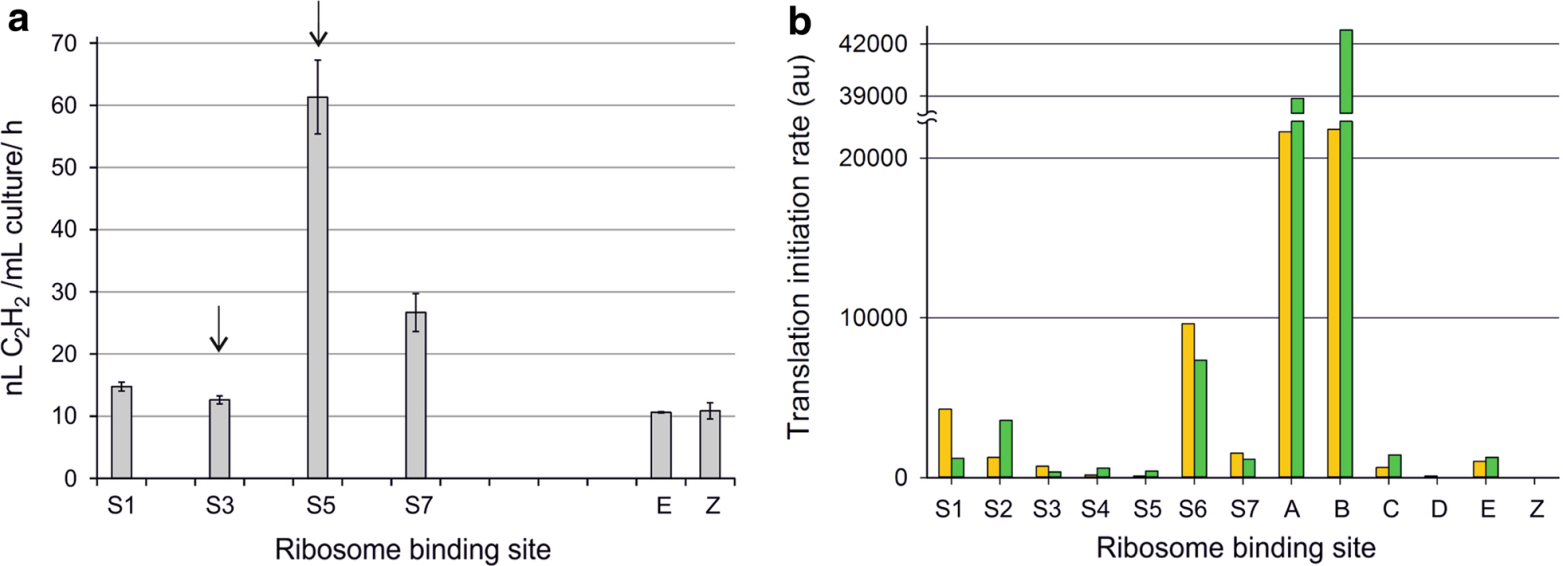
Evaluation of the translational efficiencies of selected RBS sequences based on **a** ethylene productivity of six designed strains expressing *ethylene*-*forming enzyme* measured from the headspace at 4 h after induction. The bars showing the average ethylene productivity (nL C_2_H_2_/ml culture/h) and standard deviations were calculated from three biological replicates (n = 3) and normalized to OD_750 nm_. The arrows indicate the RBSs which differ the most in comparison to the fluorescent datasets in Fig. 1. **b** Salis calculator prediction for the translation initiation rate using the nucleotide sequence around the start codon (− 25 to + 35) as input, displaying predicted translation initiation rates for all the RBSs in combination with the two fluorescent reporters sYFP2 (yellow) and GFPmut3b (green)

To further assess the connection between gene sequence and translation efficiency, six RBSs (S1, S3, S5, S7, E, Z) with relatively uniform expression profiles between YFP (Fig. 1a) and GFP (Fig. 1b) were selected for an independent analysis using *ethylene*-*forming enzyme* (E.C. 1.14.11.34) as an alternative quantitative reporter. This enzyme (encoded by *efe* from *Pseudomonas syringae*) enables the conversion of intracellular 2-oxoglutarate into ethylene [21], which can be quantitatively measured from the headspace of sealed culture vials by gas chromatography (GC). The expression plasmids carrying the *efe* gene under the control of the six alternative RBSs were assembled the same way as the fluorescent reporter constructs, transformed into *Synechocystis*, and verified by colony PCR (Additional file 3: C). The analysis revealed that ethylene production between parallel biological replicates was highly reproducible, and enabled RBS-specific quantitative evaluation between the strains in analogy to the use of the fluorescent reporters (Fig. 3a). While the relative signal intensities measured for most RBSs followed the trends observed for YFP and GFP (Fig. 1), the performance of S3 was especially poor with *efe*. In parallel, the relative activity of S5 appeared higher than expected based on YFP and GFP profiles used for the comparison.

### Sequence analysis of the RBSs fails to predict performance in *Synechocystis*

Based on the obtained activity data, the recorded efficiencies of the RBSs were complemented by in silico analysis to pinpoint potential nucleotide sequence-level determinants which would explain the most significant RBS-specific variation between the datasets. Specifically, the objective was to find correlation between putative mRNA secondary structure formation and reduced expression levels between the YFP and GFP systems for the RBSs S4, S6 and A (representing divergence) and the remaining RBSs (representing similarity). The comparison carried out with two alternative on-line prediction tools clearly revealed that the minimum free energies (MFE) calculated for the regions around the start codon (− 25 to + 35) (Additional file 6: A, B) or the entire sequences (Additional file 6: C, D) do not provide any clear indication of unfavorable sequence-level interactions which would explain the functional divergence. In addition, estimation of the translation efficiencies based on the 13 RBS sequences using the Salis calculator (Fig. 3b) and UTR Designer (Additional file 7) resulted in similar outputs that differed significantly from the measured expression profiles, supporting the view that that the reverse engineering approach does not allow relevant predictions which would correlate with the experimental findings in *Synechocystis*.

## Discussion

There is a profound need to establish new industrial solutions for sustainable large-scale production of different carbon-based chemicals to reduce our current dependency on petroleum-derived products. In this context, photosynthetic microorganisms have already been recognized at EU-level as part of emerging future technologies [22], as potential biotechnological hosts for generating desired target chemicals directly from atmospheric CO_2_. This study addressed the apparent need for more robust molecular biology tools and identification of the associated bottlenecks in cyanobacterial engineering, which have been recognized as major limitations in the development of commercially viable systems [22]. Specifically, the work focused on the systematic functional comparison of ribosome binding sites in the cyanobacterial host *Synechocystis* sp. PCC 6803, with the objective to expand the prospects for targeted use of RBSs as translational regulatory elements in sophisticated rational pathway design. From engineering viewpoint, the information provided in this study sets a basis for regulating the relative expression levels of individual genes in polycistronic operons in *Synechocystis*, which is essential for the functional optimization of heterologous pathways in future applications, as simple expression level maximization does not necessarily ensure the most efficient flux through the subsequent enzyme-catalyzed steps [3]. In addition to providing dynamic data on the function of 13 selected RBSs in vivo, the work underlines the severe constraints related to gene-specific interactions, and the importance of finding efficient strategies for expression construct assembly to meet the demands for increased complexity, predictability and preparative throughput.

Cyanobacterial engineering has traditionally relied on conventional cloning strategies which are relatively rigid for generating multiple parallel variations of constructs, or multi-gene operons which require the assembly of many different components in alternate arrangements. In addition, due to the lack of congruence between the approaches and a limited palette of validated species-specific genetic elements, finding reliable comparable information on the most the optimal components may be challenging. To overcome the preparative constraints and facilitate the design phase, we adapted a modular cloning strategy, in which compatible genetic elements can be fused together in any order via iterative subcloning steps based on restriction site recycling [3]. Besides enabling the assembly of 32 alternative *Synechocystis*-compatible reporter constructs with relative ease, the system can now be used for fusing the evaluated RBS sequences in the library with any target gene of choice, and further, allows the individual RBS-gene fragments to be joined into polycistronic operons. Importantly, the generated library together with the construction platform provides the means for pathway optimization in *Synechocystis*, as the expression level of individual proteins can now be modulated at translational level by selecting appropriate combinations of RBSs in front of the genes. Besides the rational approach, the system also is suitable for randomized RBS selection [3], which may be used for optimizing multi-gene operons even if the relative efficiency of the RBSs isn’t precisely known, yet requires access to a high-throughput screening system to identify the best-performing clones. For the future development in the field, it is also important not to be confined to a single cyanobacterial strain, and to be able to apply the developed synthetic biology tools for alternate hosts such as *Synechococcus elongatus* PCC 7942 or *Synechococcus* sp. PCC 7002 which may be better suited for a specific purpose. As demonstrated in the current work, the assembly system can be adapted for any prokaryotic expression system, simply by using a suitable host-specific vector at the last subcloning step.

Based on the acquired data from multiple parallel replicates and independently produced datasets, the results were highly reproducible, and allowed direct quantitative comparison of the translation efficiencies of the different RBS sequences. As the primary observation, the 13 characterized RBSs produced a relatively broad range of expression levels for both fluorescent reporters (Fig. 1), thus allowing access to dynamic translation-level regulation in engineered systems in *Synechocystis*. As a downside from engineering viewpoint, however, the nucleotide sequence of the target gene has potentially a profound effect on the expression level, as seen in the low correlation between the signal profiles produced by YFP and the codon-optimized GFP (Table 4). The observation is not unexpected as such, and the phenomenon has been encountered earlier in cyanobacteria, but up until now the extent to which this may interfere with the function of engineered cyanobacterial expression systems has remained elusive. Generally, the effect is caused by mRNA secondary structure formation between a specific RBS and the coding region sequence, which may affect the ribosome recognition or efficient initiation complex formation. Two of the RBSs which functioned in the most unpredictable manner in the test set-up (Fig. 1; see arrows) are S4 from *Synechocystis psbA2* gene encoding the photosystem II reaction center protein D1, which has been used in many engineering applications, and A which has been reported to give the highest translation levels in *E. coli* [3, 12]. While the results show that S4 and A would not have been optimal choices for maximizing the expression of the YFP and GFP, respectively, it is recognized that the interactions are in each case determined by the associated combined effects with the following gene sequence and not the specific RBS sequences per se. This was also corroborated in the independent analytical trial where the RBS S3 from *cpcB* encoding the phycocyanin subunit β—one of the most efficient RBSs in *Synechocystis* [23] used in a number of expression systems—functioned very poorly in context with an alternate heterologous reporter gene *efe* (Fig. 3a). The complication is further emphasized by the inability of currently available in silico prediction tools to approximate the relative RBS efficiencies for *Synechocystis* (Fig. 3b and Additional file 7) or to identify potentially interfering mRNA secondary structures in specified RBSs which perform in a context-dependent manner in the set-up (Additional file 6). This discrepancy is expected to result from, at least in part, a longer optimal distance between the SD core sequence and the start codon, as well as the organism-specific culture conditions such as growth temperature, between *Synechocystis* and *E. coli* for which the system has been optimized for [24]. However, a significant increase was observed in the correlation between the recorded YFP and GFP full fluorescence datasets in response to the exclusion of the most inconsistent RBSs from the comparison (Table 4), which indicates that (i) the expression profiles are not random but (ii) clearly follow an underlying pattern which may be masked by gene-specific interactions. This suggests that the obtained datasets can be used to a certain extent to predict the relative expression efficiencies (i.e. evaluating the *potential*), with the caution that the true performance of a specific RBS with a given gene would also require experimental verification. Although exact numerical comparison may be meaningless due to the observed context-dependence, the RBSs can be at least roughly divided into groups based on apparent high, moderate and low activity, which may serve as an initial guideline for selecting appropriate control elements for engineering work.

As seen from the combined YFP and GFP data, the RBSs S3, S4 and A (Fig. 1 and Table 2) appear to have *potential* as prominent high-activity elements useful for maximizing translational efficiency in *Synechocystis.* This is not unexpected since S3 (*cpcB*) and S4 (*psbA2*) derive from genes which are exceptionally highly expressed in the native context, while A performs at the most optimal efficiency in heterologous systems in *E. coli*. The remaining cyanobacterial RBSs (or RBSs used in cyanobacteria), S1, S2, S5 and S7 (Fig. 1 and Table 2), displayed activities between 25 and 50% of the maximum capacity in the experimental set-up. These efficiencies appear relatively moderate, considering that S1 represents a sequence specifically optimized for the host [4, 25], S2 originates from the homologous *cpcB* gene in *Synechococcus* sp. PCC 7002, and S5 is from *rbcL* encoding for the large subunit of Rubisco in *Synechocystis*, but again, may suffer from the context-dependent effects. RBS S7 from the expression plasmid pDF-lac [20], which has been applied in various studies in *Synechocystis* with generally high expression profiles, performed at about half of the maximum activity for all the three reporter systems. The RBSs A–Z spanned the entire activity range and roughly followed the order as reported for *E. coli*, although gene-specific deviation could be observed (Fig. 1). In order to employ the available potential of using the RBSs for reliable translational tuning in *Synechocystis*, it is clear that that new approaches are needed to decouple UTR region and coding sequence interactions. One possible strategy would be to design additional downstream insulator sequences to minimize potential interfering secondary structure formation between the RBS and the immediate downstream region [26] to reduce the observed gene-dependent effects in cyanobacterial expression systems.

## Conclusions

A set of 13 different RBS sequences characterized in this work can be used for the regulation of the protein expression efficiency at a wide dynamic range in the cyanobacterium *Synechocystis* sp. PCC 6803. However, the absolute activity of a specific RBS is difficult to determine due to sequence-specific effects caused by the downstream target gene, which may result in significantly decreased translation efficiencies. Despite this context dependence, there appears to be a level of similarity between the underlying expression profiles, which can be used as an indicator of the potential performance of the alternative RBSs in engineered systems. Due to the limited availability of associated functional information in cyanobacteria, reliable prediction of potential interactions that may reduce translation efficiency is difficult, and calls for new design strategies to minimize unwanted interactions between the RBS and the downstream target gene.

## Methods

### Enzymes and reagents

The restriction enzymes, T4 DNA ligase and DNA polymerase used in this work were purchased from New England BioLabs (USA) or from ThermoFischer-Scientific (USA). Commercial Qiagen (Germany) kits were used for plasmid isolation (QIAprep Spin miniprep kit) and gel extraction (QIAquick, gel extraction kit). Oligonucleotides were ordered from Eurofins MWG Operon (Germany), and larger gene fragments from GenScript (USA). All other chemicals were purchased from Sigma-Aldrich (USA), unless mentioned otherwise.

### Bacterial strains and standard growth conditions

*Escherichia coli* strain DH5α was used for plasmid propagation and selection in the preparative molecular biology steps. The cells were grown in Luria–Bertani (LB) medium at 37 °C in a shaker at 150–200 rpm or on the solid LB plates containing 1.5% (w/v) agar. When necessary, LB medium was supplemented with appropriate antibiotics at concentrations 50 µg/ml spectinomycin (Sp) and 34 µg/ml chloramphenicol (Cm).

A glucose tolerant substrain of *Synechocystis* sp. PCC 6803 [27] obtained originally from Professor Aaron Kaplan (Hebrew University of Jerusalem, IL) was used for all the cyanobacterial experiments. The cells were grown in 25–50 ml Erlenmeyer flasks in liquid BG-11 medium buffered with 20 mM TES-KOH (pH 8.0) [28] with supplemented 25 µg/ml Sp and 10 µg/ml Cm to maintain transformant selection pressure throughout all cultivations. The cultures were incubated at 30 °C in ~ 120 rpm orbital shaking under continuous light of 20–50 μmol photons m^−2^ s^−1^ under 1% CO_2_ atmosphere (MLR-351 growth chamber Sanyo, Japan) or under ambient CO_2_ (Algaetron 230 growth chamber, Photon Systems Instruments, CZ). Solid plate cultivations were conducted on BG-11 plates containing additional 1.5% (w/v) Bactoagar (Difco, USA) and 0.3% (w/v) sodium thiosulfate under corresponding conditions (MLR-351, Sanyo).

### Generating the RBS library

Six of the RBS fragments used in the study (A–E, Z) (Table 2) were provided by Professor Ron Milo as corresponding pNiv constructs [3]. The remaining RBS fragments were designed to carry a 22 bp variable region immediately upstream the start codon, corresponding to the exact native target gene sequences acquired from NCBI GenBank (S2–S5) or existing expression construct sequences (S1, S6–S7) (Table 2), and preceded by a 27 bp invariable upstream insulator sequence common to all the constructs (5′-TAATAGAAATAATTTTGTTTAACTTTA-3′). These fragments were ordered as synthesized complementary single-stranded oligonucleotides (MWG, Germany), mixed in pairs, and subcloned into pNiv (*Spe*I-*Nsi*I). The resulting library was verified by sequencing using the primer 5′-CTTCCTGTTAGTTAGTTACTTAAGCTCGG -3′.

### Generation of the reporter genes

The three reporter genes, sYFP2 [18], GFPmut3b [19] and *efe* [21] used in the study were ordered as synthetic fragments (GenScipt). The genes for sYFP2 (GenBank ID DQ092361.1) and *efe* (GenBank ID AF101058.1) were based on the original NCBI nucleotide sequences, while GFPmut3b (GenBank ID AAB51348.1) was codon-optimized for *Synechosystis* (Additional file 1). The fragments were designed as chloramphenicol resistance cassette (Cm^R^) fusions [3] flanked by restriction sites *Nsi*I and *Xho*I, and avoiding the restriction sites *Eco*RI, *Spe*I, *Nhe*I, and *Sal*I within the coding regions to ensure compatibility with the assembly system [3].

### Assembly of the RBS-reporter constructs

The construct assembly system adapted for fusing the RBS sequences with the reporter genes was based on a modular cloning strategy described by Zelcbuch et al. [3]. The genes (synthesized as Cm^R^-fusions for selection) were first subcloned directly downstream of each of the 13 RBS sequences in the pNiv carrier plasmids (*Nsi*I-*Xho*I), followed by the transfer of the combined fragments into an expression plasmid pDF-lac2 (*Spe*I-*Sal*I) specifically designed for the purpose. All the plasmids generated in this study are listed in Additional file 2.

### Assembly of a compatible expression plasmid pDF-lac2

The expression plasmid used for characterizing the RBSs in *Synechocystis* was generated from the shuttle vector pDF-lac [20] by replacing the *Spe*I-*Sal*I region of the plasmid spanning the promoter region with a compatible synthetic *Nhe*I-*Sal*I fragment (see sequence below; restriction sites *Nhe*I, *Spe*I and *Sal*I underlined, respectively). This modification placed the *Spe*I site in between the promoter and the existing *Sal*I site, thus allowing direct transfer of fragments from the pNiv assembly plasmid (*Spe*I-*Sal*I) under the control of the promoter P_A1lacO-1_.

5′-GCTAGCGAGCGGATACATATTTGAATGTATTTAGAAAAATAAACAAATAGGGGTTCCGCGCACATTTCCCCGAAAAGTGCCACCTGACGTCTAAGAAACCATTATTATCATGACATTAACCTATAAAAATAGGCGTATCACGAGGCCCTTTCGTCTTCACCTCGAGAAATTTATCAAAAAGAGTGTTGACTTGTGAGCGGATAACAATGATACTTAGATTCAATTGTGAGCGGATAACAATTTCACACAGAATTCATTAAAGACTAGTAAGGTACCCGGGGATCCTCTAGAGTCGAC-3′.

### Generation of the *Synechocystis* strains for RBS comparison

The generated pDF-lac2—based expression constructs (Additional file 2) were transformed into the WT *Synechocystis* [29] and plated on BG11 plates supplemented with increasing amounts of Sp and Cm. Antibiotic resistant clones were transferred onto secondary plates, confirmed by colony PCR, and stored at − 80 °C with 7.5% DMSO until characterization.

### Quantitative fluorescence analysis of the *Synechocystis* strains expressing YFP and GFP

Quantitative fluorescent analysis of the *Synechocystis* strains expressing sYFP2 and GFPmut3b under the control of the alternative RBSs was carried out using a Tecan microplate reader (Tecan infinite 200 PRO) with 495 nm (ex)/535 nm (em) and 485 nm (ex)/525 nm (em), respectively. The analysis was conducted on intact cells (culture volume 150 µl) on 96-well black clear bottom polystyrene plates (Costar Corning, USA) for six biological replicates (*full dataset*) or one representative clone (1-*day dataset*) with three technical replicates in each case. To allow reproducible comparison, the pre-cultures inoculated from freshly prepared plates were first diluted to OD_750_ 0.28 (Thermo Scientific GENESYS 10S UV–Vis spectrophotometer), and grown under ambient CO_2_ for ~ 18 h. At OD_750_ ~ 0.5 the cultures were induced by the addition of 1 mM of isopropyl-β-D-thiogalactopyranoside (IPTG) and incubated further under 1% CO_2_ until analysis (alongside uninduced controls). The fluorescence was measured at time points 2, 4, and 6 h after induction using 25 flashes with nine reads per well, normalized to cell density (Tecan infinite 200 PRO), and represented as relative values to allow more convenient comparison between the YFP and GFP datasets.

### Ethylene quantitation

Ethylene production efficiency of the constructed *Synechocystis* strains was monitored by quantitating ethylene from the headspace of sealed culture vials by GC against a commercial gas standard (AGA; 99% N_2_, 1% C_2_H_2_ v/v [20]. In each case, three biological replicates were sampled at 4 h after induction using GC-FID (Perkin Elmer AutoSystem) with CP-CarboBOND fused silica capillary column (Varian, 50 m × 0.53 mm) under isothermal conditions (oven and injector 80 °C, and detector 200 °C with H_2_ carrier gas at a flow rate 7 ml min^−1^). To allow direct comparison between the samples, ethylene productivity was normalized against culture optical density (750 nm) for calculating average values and corresponding standard deviations.

### Statistical comparison of the datasets

Statistical correlation between the different datasets was evaluated by using two alternative approaches, Pearson correlation coefficient [30] and Spearman’s rank correlation [31, 32]. The correlation coefficients and the corresponding p values were calculated in each case from the averaged data using XLSTAT plugin in Microsoft Excel.

### Sequence analysis

Nucleotide sequence analysis of the alternative RBSs in context with the YFP and GFP was performed using the Salis RBS calculator [12] and the UTR Designer [33] for the prediction of the translation initiation rate, using the − 25 to + 35 sequences of the mRNA transcripts as input (reverse engineering). The same sequences, as well as the entire coding regions (− 25 until the end-codon), were also analyzed using RNAfold Server hosted by ViennaRNA web service [34–36] and mfold web server [37] to predict the most stable mRNA secondary structures with the minimum free energy (MFE).

## Additional files

**Additional file 1.** Nucleotide sequence of the synthetic GFPmut3b gene codon optimized for *Synechosystis* sp. PCC 6803.

**Additional file 2.** List of the expression plasmid constructs generated in this study for evaluating the performance of 13 selected RBS in *Synechocystis* sp. PCC 6803, using sYFP2, GFPmut3 and *efe* as quantitative reporters.

**Additional file 3.** Colony PCR verification of the *Synechocystis* sp. PCC 6803 strains generated in this study as visualized by agarose gel electrophoresis.

**Additional file 4.** The full fluorescence activity profiles of the generated *Synechocystis* sp. PCC 6803 strains expressing sYFP2 and GFPmut3b under the control of the 13 selected RBSs (0–6 h).

**Additional file 5.** Growth of the *Synechocystis* sp. PCC 6803 strains expressing sYFP2 under the control of the 13 selected RBS in comparison to the WT control strain (OD _750 nm_ at time-points 0, 6 and 24 h).

**Additional file 6.** mRNA secondary structure minimum free energy values calculated using RnaFold server and mfold web server prediction tool for the 13 different RBS sequences in context with sYFP2 and GFPmut3b.

**Additional file 7.** Expression levels predicted by UTR Designer for sYFP2 and GFPmut3b in combination with the 13 target RBS sequences used in the study.

## Abbreviations

Bp: base pairs
Cm: chloramphenicol
D: day
*E. coli*: *Escherichia coli*
Em: emission
Ex: exitation
GC: gas chromatography
GFP: GFPmut3b
IPTG: isopropyl-β-d-1-thiogalactopyranoside
LB: Luria–Bertani
MFE: minimum free energy
OD: optical density
ORF: open reading frame
RBS: ribosome binding site
Rpm: revolutions per minute
SD: Shine-Dalgarno
Sp: spectinomycin
*Synechocystis*: *Synechocystis* sp. PCC 6803
WT: wild type
YFP: sYFP2
