# Supplementary material for: Translation efficiency of heterologous proteins is significantly affected by the genetic context of RBS sequences in engineered cyanobacterium Synechocystis sp. PCC 6803
Source: Microb Cell Fact. 2018 Mar 2;17:34. doi: 10.1186/s12934-018-0882-2 (PMC5834881; doi:10.1186/s12934-018-0882-2)
Supplement: Supplementary file 1 — Additional file 1. Nucleotide sequence of the synthetic GFPmut3b gene codon optimized for Synechosystis sp. PCC 6803. [file 12934_2018_882_MOESM1_ESM.pdf]

ATGCATAGCAAAGGTGAAGAACTGTTTACTGGAGTGGTGCCCATTCTGGTTGAACTGGACGGCGATGTGAACGGTCATAAATT
TAGTGTTAGCGGCGAAGGTGAAGGGGATGCCACCTACGGCAAATTGACCTTGAAATTTATTTGTACCACCGGTAAATTGCCCG
TGCCCTGGCCCACCTTAGTGACCACCTTTGGCTATGGTGTGCAATGTTTTGCCCGTTATCCCGATCATATGAAACAACATGAT
TTCTTTAAAAGTGCCATGCCCGAAGGTTATGTGCAAGAACGGACCATTTTCTTTAAAGATGATGGGAACTACAAAACCCGCGC
CGAAGTGAAATTTGAAGGGGATACCTTGGTGAATCGTATTGAATTGAAAGGCATTGATTTTAAAGAAGATGGTAATATTTTGG
GGCATAAATTAGAATACAACTACAACAGTCATAATGTGTATATTATGGCCGATAAACAGAAAAATGGCATTAAAGTGAACTTT
AAAATTCGGCATAACATTGAAGATGGTTCCGTGCAATTGGCCGATCATTATCAACAAAATACCCCCATTGGGGATGGCCCCGT
GTTGTTACCCGATAATCATTATTTGTCCACCCAAAGTGCCTTATCCAAAGATCCCAATGAAAAACGCGATCATATGGTGCTGT
TAGAATTTGTTACCGCTGCTGGGATTACCCACGGAATGGACGAATTATACAAATAAGCTAGCGTTGATCGGGCACGTAAGAGG
TTCCAACTTTCACCATAATGAAATAAGATCACTACCGGGCGTATTTTTTGAGTTATCGAGATTTTCAGGAGCTAAGGAAGCTA
AAATGGAGAAAAAAATCACGGGATATACCACCGTTGATATATCCCAATGGCATCGTAAAGAACATTTTGAGGCATTTCAGTCA
GTTGCTCAATGTACCTATAACCAGACCGTTCAGCTGGATATTACGGCCTTTTTAAAGACCGTAAAGAAAAATAAGCACAAGTT
TTATCCGGCCTTTATTCACATTCTTGCCCGCCTGATGAACGCTCACCCGGAGTTTCGTATGGCCATGAAAGACGGTGAGCTGG
TGATCTGGGATAGTGTTCACCCTTGTTACACCGTTTTCCATGAGCAAACTGAAACGTTTTCGTCCCTCTGGAGTGAATACCAC
GACGATTTCCGGCAGTTTCTCCACATATATTCGCAAGATGTGGCGTGTTACGGTGAAAACCTGGCCTATTTCCCTAAAGGGTT
TATTGAGAATATGTTTTTTGTCTCAGCCAATCCCTGGGTGAGTTTCACCAGTTTTGATTTAAACGTGGCCAATATGGACAACT
TCTTCGCCCCCGTTTTCACGATGGGCAAATATTATACGCAAGGCGACAAGGTGCTGATGCCGCTGGCGATCCAGGTTCATCAT
GCCGTTTGTGATGGCTTCCATGTCGGCCGCATGCTTAATGAATTACAACAGTACTGTGATGAGTGGCAGGGCGGGGCGTAATA
AGCTAGCGCGGCCGCTCGAG

Additional file 1: Nucleotide sequence of the synthetic GFPmut3b gene codon optimized for *Synechosystis* sp. PCC 6803. The gene sequence is shown in black, chloramphenicol cassette in grey and the restriction sites in red font.
