# Supplementary material for: Translation efficiency of heterologous proteins is significantly affected by the genetic context of RBS sequences in engineered cyanobacterium Synechocystis sp. PCC 6803
Source: Microb Cell Fact. 2018 Mar 2;17:34. doi: 10.1186/s12934-018-0882-2 (PMC5834881; doi:10.1186/s12934-018-0882-2)
Supplement: Supplementary file 2 — Additional file 2. List of the expression plasmid constructs generated in this study for evaluating the performance of 13 selected RBS in Synechocystis sp. PCC 6803, using sYFP2, GFPmut3 and efe as quantitative reporters. [file 12934_2018_882_MOESM2_ESM.pdf]

Additional file 2. The expression constructs generated in this study for the evaluation of different RBS sequences in respect to translational efficiency in *Synechocystis* sp. PCC 6803, using sYFP2, GFPmut3 and *efe* as alterative quantitative reporters. See Table 2 for the nucleotide sequences of the RBSs.

**RBS**	**Expression construct name**		
	**sYFP2**	**GFPmut3b**	**efe**
S1	pNiv(S1)-sYFP2-CmR	pDF-lac2-(S1)-GFPmut3b-CmR	pDF-lac2-(S1)-efe-CmR
S2	pNiv(S2)-sYFP2-CmR	pDF-lac2-(S2)-GFPmut3b-CmR	
S3	pNiv(S3)-sYFP2-CmR	pDF-lac2-(S3)-GFPmut3b-CmR	pDF-lac2-(S3)-efe-CmR
S4	pNiv(S4)-sYFP2-CmR	pDF-lac2-(S4)-GFPmut3b-CmR	
S5	pNiv(S5)-sYFP2-CmR	pDF-lac2-(S5)-GFPmut3b-CmR	pDF-lac2-(S5)-efe-CmR
S6	pNiv(S6)-sYFP2-CmR	pDF-lac2-(S6)-GFPmut3b-CmR	
S7	pNiv(S7)-sYFP2-CmR	pDF-lac2-(S7)-GFPmut3b-CmR	pDF-lac2-(S7)-efe-CmR
A	pNiv(A)-sYFP2-CmR	pDF-lac2-(A)-GFPmut3b-CmR	
B	pNiv(B)-sYFP2-CmR	pDF-lac2-(B)-GFPmut3b-CmR	
C	pNiv(C)-sYFP2-CmR	pDF-lac2-(C)-GFPmut3b-CmR	
D	pNiv(D)-sYFP2-CmR	pDF-lac2-(D)-GFPmut3b-CmR	
E	pNiv(E)-sYFP2-CmR	pDF-lac2-(E)-GFPmut3b-CmR	pDF-lac2-(E)-efe-CmR
Z	pNiv(Z)-sYFP2-CmR	pDF-lac2-(Z)-GFPmut3b-CmR	pDF-lac2-(Z)-efe-CmR
