# Supplementary material for: Translation efficiency of heterologous proteins is significantly affected by the genetic context of RBS sequences in engineered cyanobacterium Synechocystis sp. PCC 6803
Source: Microb Cell Fact. 2018 Mar 2;17:34. doi: 10.1186/s12934-018-0882-2 (PMC5834881; doi:10.1186/s12934-018-0882-2)
Supplement: Supplementary file 3 — Additional file 3. Colony PCR verification of the Synechocystis sp. PCC 6803 strains generated in this study as visualized by agarose gel electrophoresis. [file 12934_2018_882_MOESM3_ESM.pdf]

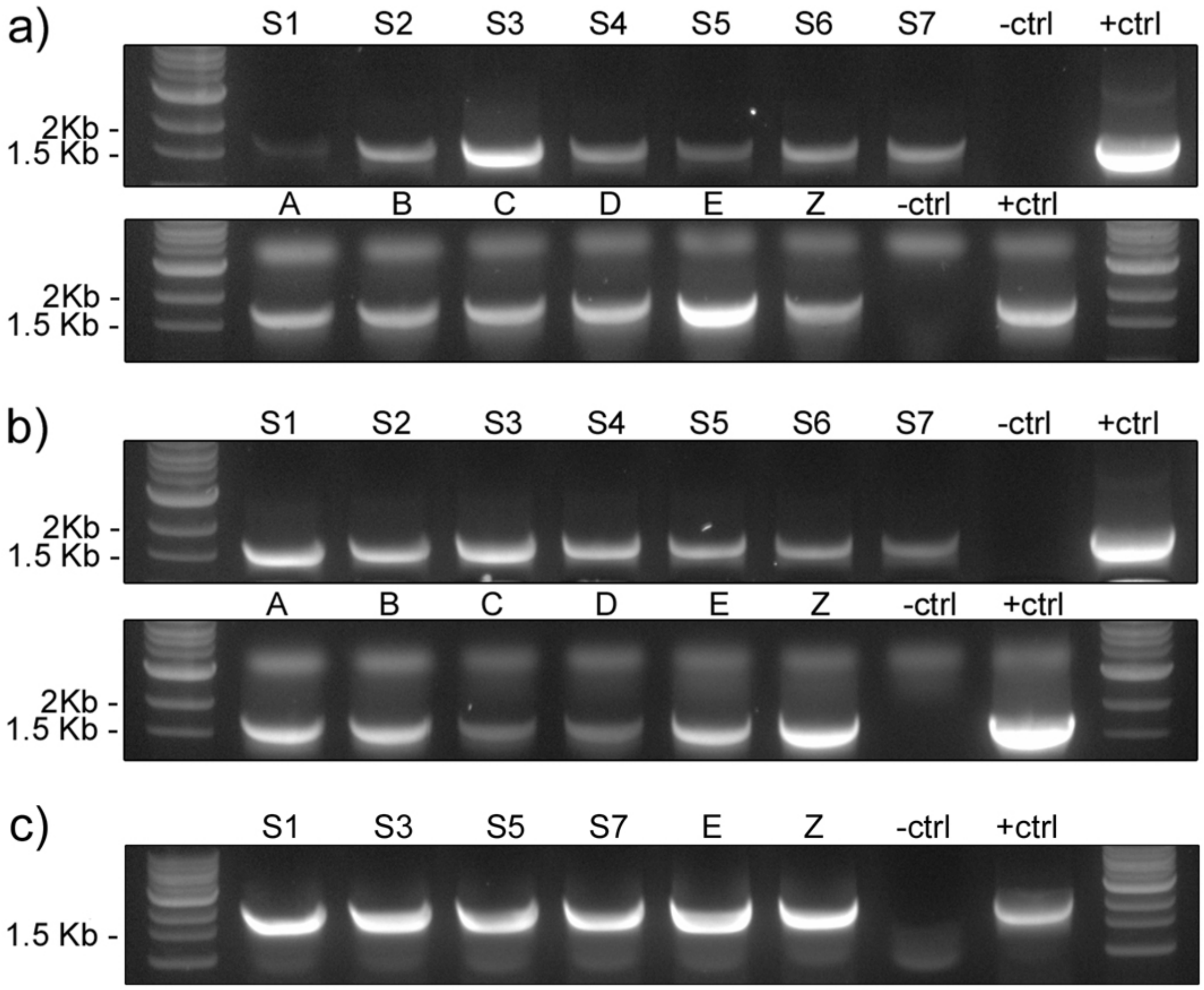

Additional file 3: Colony PCR verification of the generated *Synechocystis* sp. PCC 6803 strains as visualized by agarose gel electrophoresis. The strains harboring the (A) sYFP2 construct (expected band size ~1.5 Kb), (B) GFPmut3b construct (expected band size ~1.5 Kb), and (C) *efe* (expected band size ~1.5 Kb). The plasmids pDF-lac2-S1-sYFP2-Cmr, pDF-lac2-S1-GFPmut3b-Cmr and pDF-lac2-S1-efe-Cmr, have been used as positive controls, respectively.
