# Supplementary material for: Translation efficiency of heterologous proteins is significantly affected by the genetic context of RBS sequences in engineered cyanobacterium Synechocystis sp. PCC 6803
Source: Microb Cell Fact. 2018 Mar 2;17:34. doi: 10.1186/s12934-018-0882-2 (PMC5834881; doi:10.1186/s12934-018-0882-2)
Supplement: Supplementary file 4 — Additional file 4. The full fluorescence activity profiles of the generated Synechocystis sp. PCC 6803 strains expressing sYFP2 and GFPmut3b under the control of the 13 selected RBSs (0–6 h). [file 12934_2018_882_MOESM4_ESM.pdf]

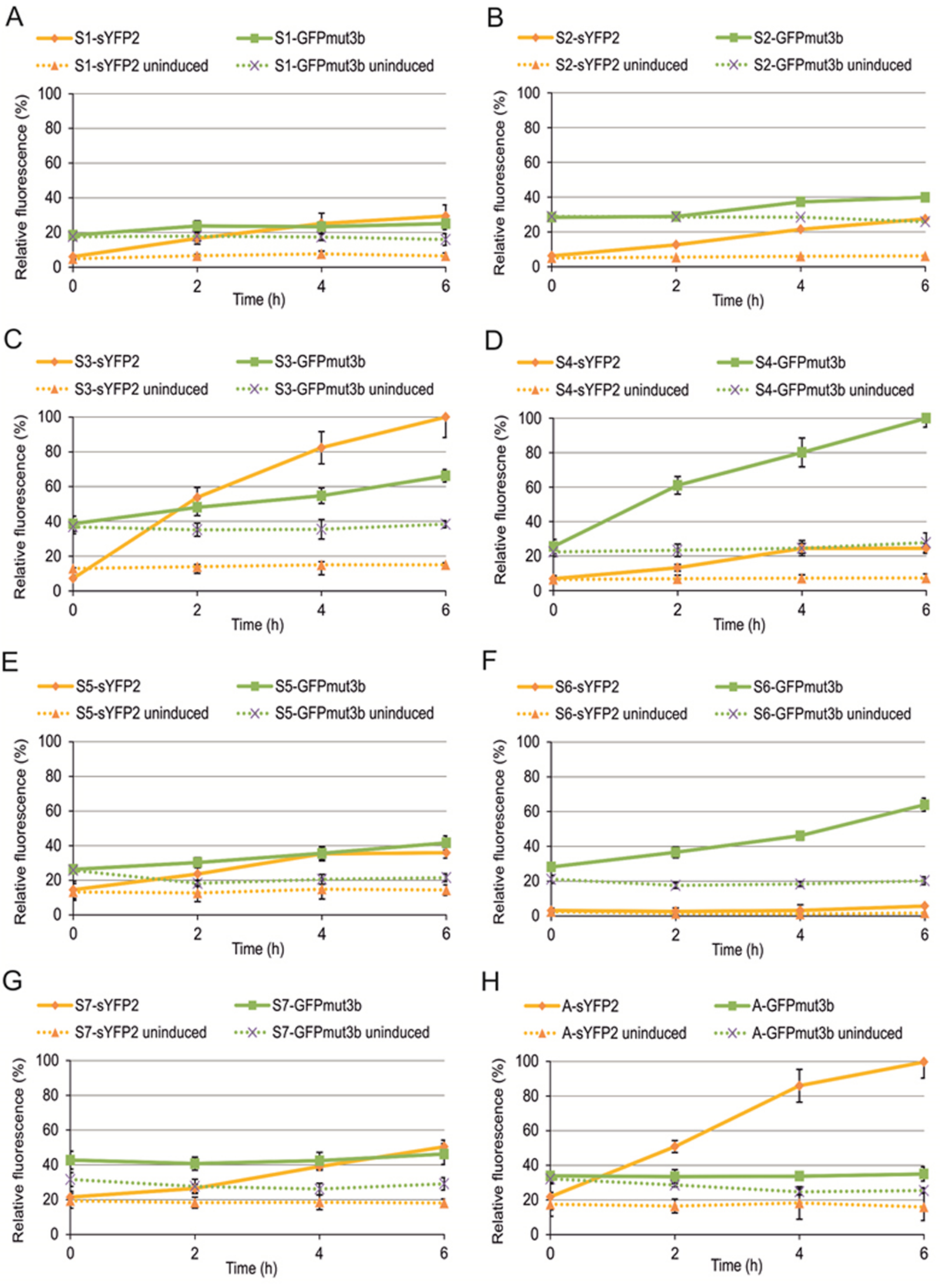

A
S1-sYFP2
S1-GFPmut3b
S1-sYFP2 uninduced
S1-GFPmut3b uninduced
Relative fluorescence (%)
Time (h)
B
S2-sYFP2
S2-GFPmut3b
S2-sYFP2 uninduced
S2-GFPmut3b uninduced
Relative fluorescence (%)
Time (h)
C
S3-sYFP2
S3-GFPmut3b
S3-sYFP2 uninduced
S3-GFPmut3b uninduced
Relative fluorescence (%)
Time (h)
D
S4-sYFP2
S4-GFPmut3b
S4-sYFP2 uninduced
S4-GFPmut3b uninduced
Relative fluorescne (%)
Time (h)
E
S5-sYFP2
S5-GFPmut3b
S5-sYFP2 uninduced
S5-GFPmut3b uninduced
Relative fluorescence (%)
Time (h)
F
S6-sYFP2
S6-GFPmut3b
S6-sYFP2 uninduced
S6-GFPmut3b uninduced
Relative fluorescence (%)
Time (h)
G
S7-sYFP2
S7-GFPmut3b
S7-sYFP2 uninduced
S7-GFPmut3b uninduced
Relative fluorescence (%)
Time (h)
H
A-sYFP2
A-GFPmut3b
A-sYFP2 uninduced
A-GFPmut3b uninduced
Relative fluorescence (%)
Time (h)

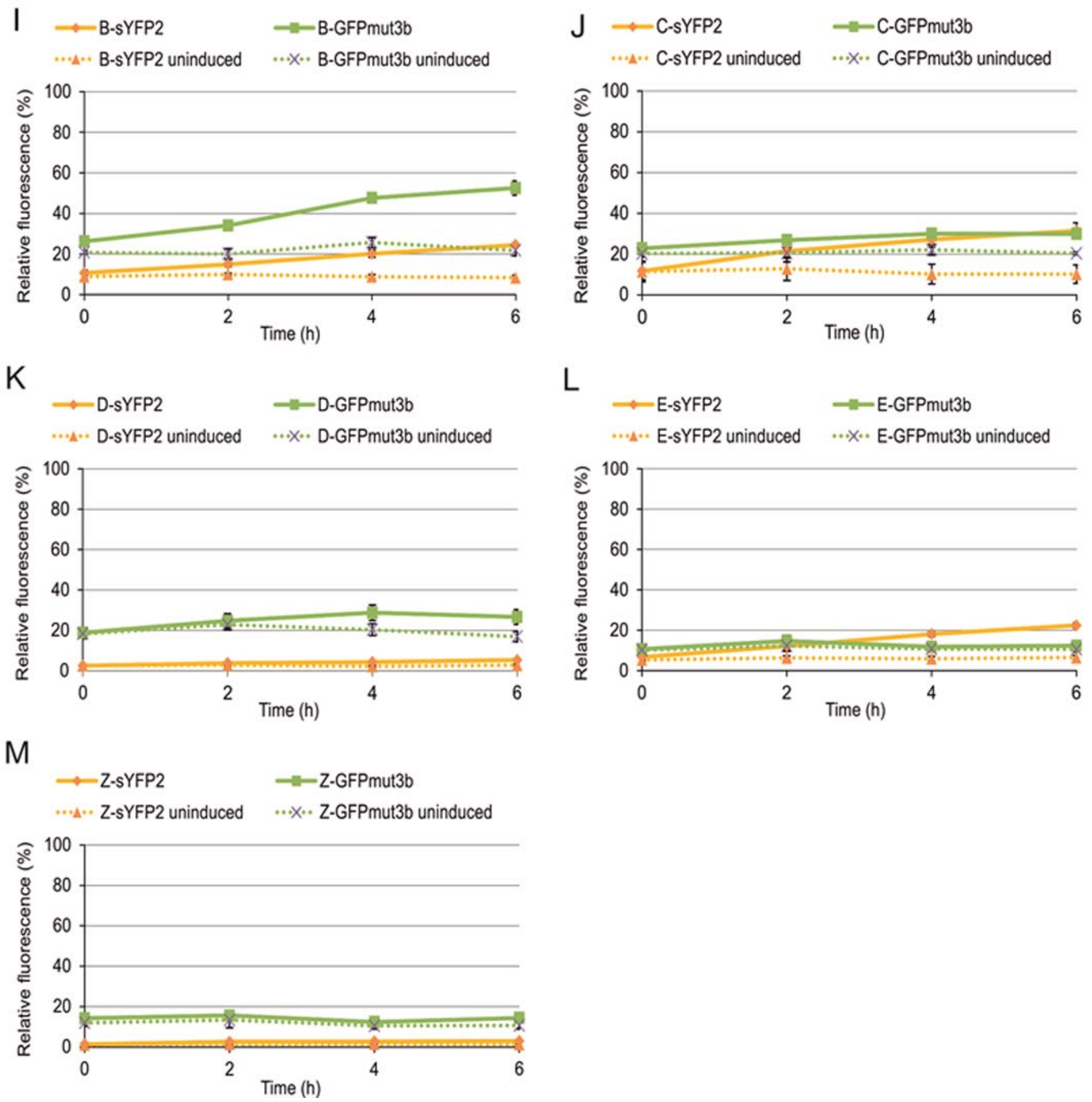

Additional file 4: Time course analysis of the generated *Synechocystis* sp. PCC 6803 strains expressing sYFP2 and GFPmut3b under the control of the 13 selected RBSs, subjected to fluorescence measurements between 0 - 6h after induction. The yellow and green lines represent the strains expressing sYFP2 and GFPmut3b, respectively. Solid lines depict induced samples and dotted lines uninduced samples. The data points have been calculated from the six parallel biological replicates with three technical replicates each (n=18).
