# Supplementary material for: Translation efficiency of heterologous proteins is significantly affected by the genetic context of RBS sequences in engineered cyanobacterium Synechocystis sp. PCC 6803
Source: Microb Cell Fact. 2018 Mar 2;17:34. doi: 10.1186/s12934-018-0882-2 (PMC5834881; doi:10.1186/s12934-018-0882-2)
Supplement: Supplementary file 5 — Additional file 5. Growth of the Synechocystis sp. PCC 6803 strains expressing sYFP2 under the control of the 13 selected RBS in comparison to the WT control strain (OD 750 nm at time-points 0, 6 and 24 h). [file 12934_2018_882_MOESM5_ESM.pdf]

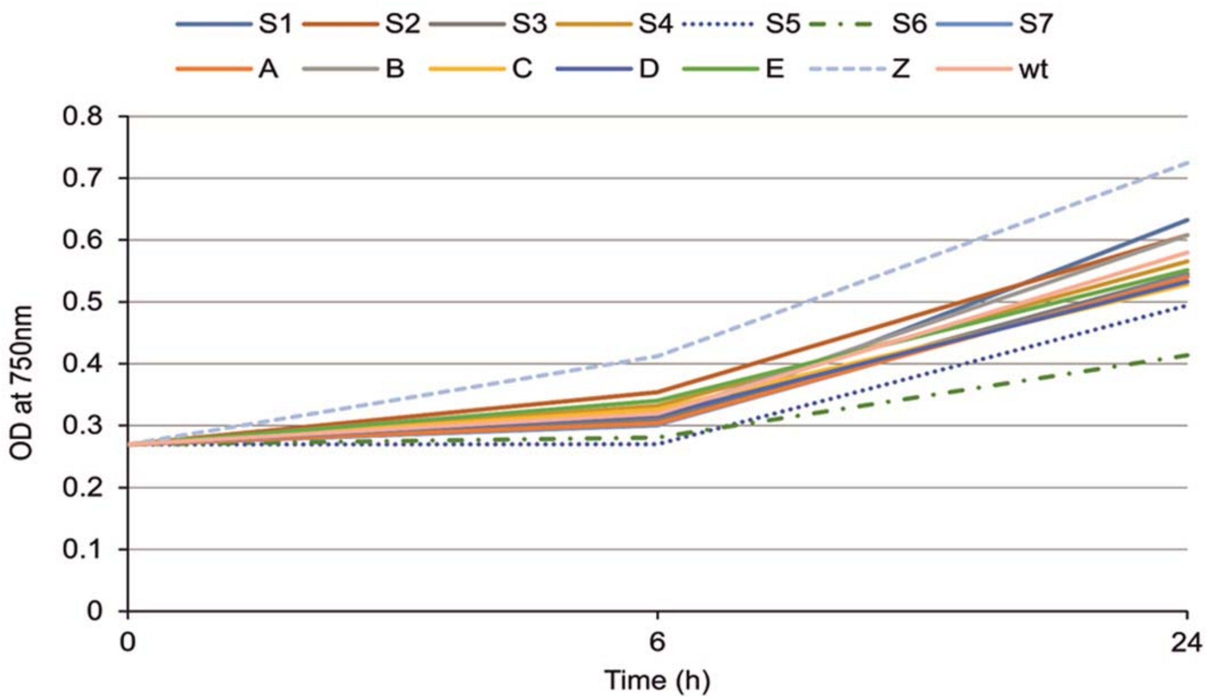

Additional file 5: Growth of the *Synechocystis* sp. PCC 6803 strains expressing sYFP2 under the control of the 13 selected RBS, in comparison to the WT control strain. The growth was monitored as OD $_{750nm}$ at time-points 0 h, 6 h and 24 h after induction with the microplate reader (Tecan infinite 200 PRO), in parallel to the fluorescence measurements.
