# Supplementary material for: Translation efficiency of heterologous proteins is significantly affected by the genetic context of RBS sequences in engineered cyanobacterium Synechocystis sp. PCC 6803
Source: Microb Cell Fact. 2018 Mar 2;17:34. doi: 10.1186/s12934-018-0882-2 (PMC5834881; doi:10.1186/s12934-018-0882-2)
Supplement: Supplementary file 6 — Additional file 6. mRNA secondary structure minimum free energy values calculated using RnaFold server and mfold web server prediction tool for the 13 different RBS sequences in context with sYFP2 and GFPmut3b. [file 12934_2018_882_MOESM6_ESM.pdf]

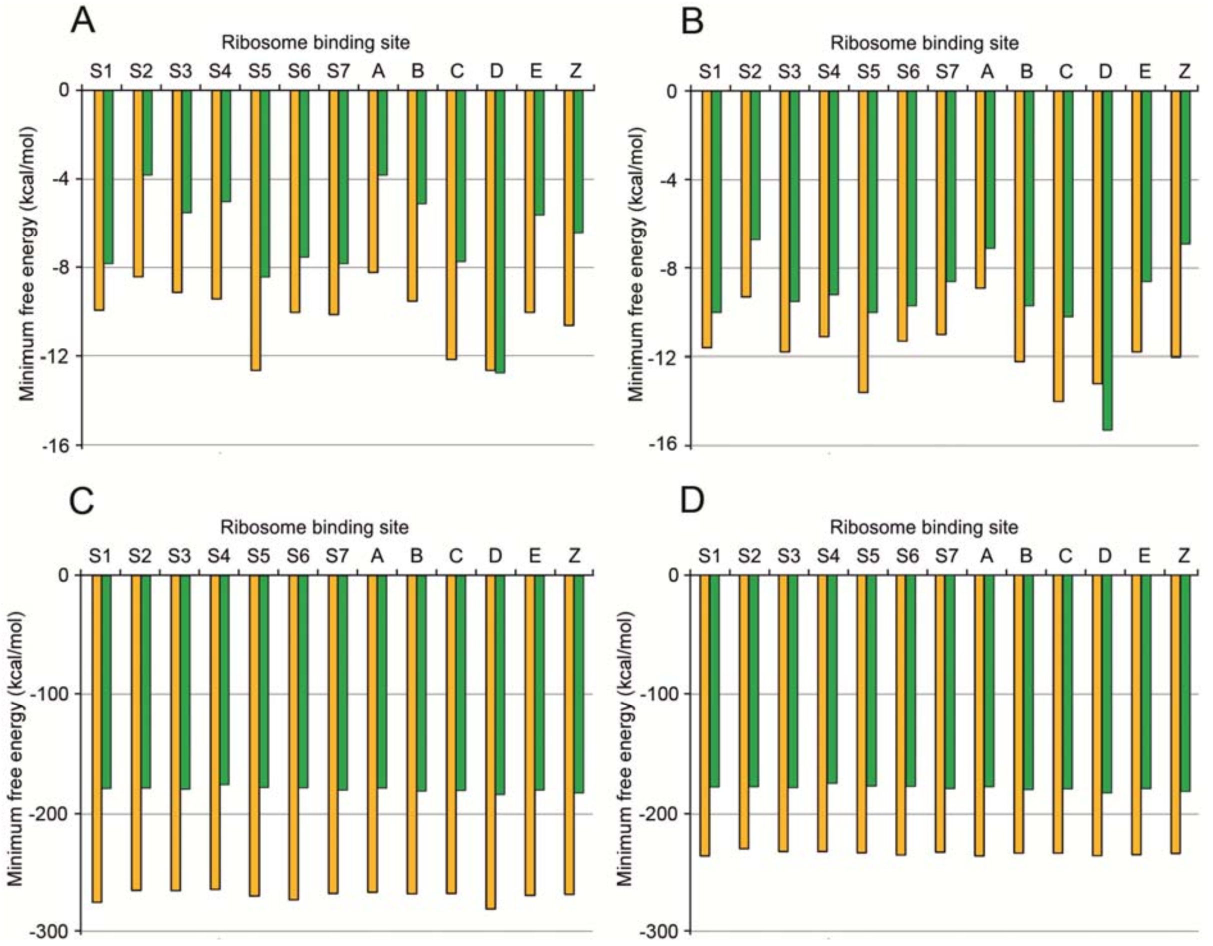

Additional file 6: The calculated minimum free energy values for the mRNA secondary structures for the 13 different RBS sequences in context with sYFP2 (yellow) and GFPmut3b (green). The values were calculated by (A) RnaFold server and (B) mfold web server prediction tool using the nucleotide sequence around the start codon (-25 to +35) as input, and in parallel, using (C) RnaFold server and (D) mfold web server prediction tool using the entire sequence (from -25 to the end of the gene) as input.
