# Supplementary material for: Translation efficiency of heterologous proteins is significantly affected by the genetic context of RBS sequences in engineered cyanobacterium Synechocystis sp. PCC 6803
Source: Microb Cell Fact. 2018 Mar 2;17:34. doi: 10.1186/s12934-018-0882-2 (PMC5834881; doi:10.1186/s12934-018-0882-2)
Supplement: Supplementary file 7 — Additional file 7. Expression levels predicted by UTR Designer for sYFP2 and GFPmut3b in combination with the 13 target RBS sequences used in the study. [file 12934_2018_882_MOESM7_ESM.pdf]

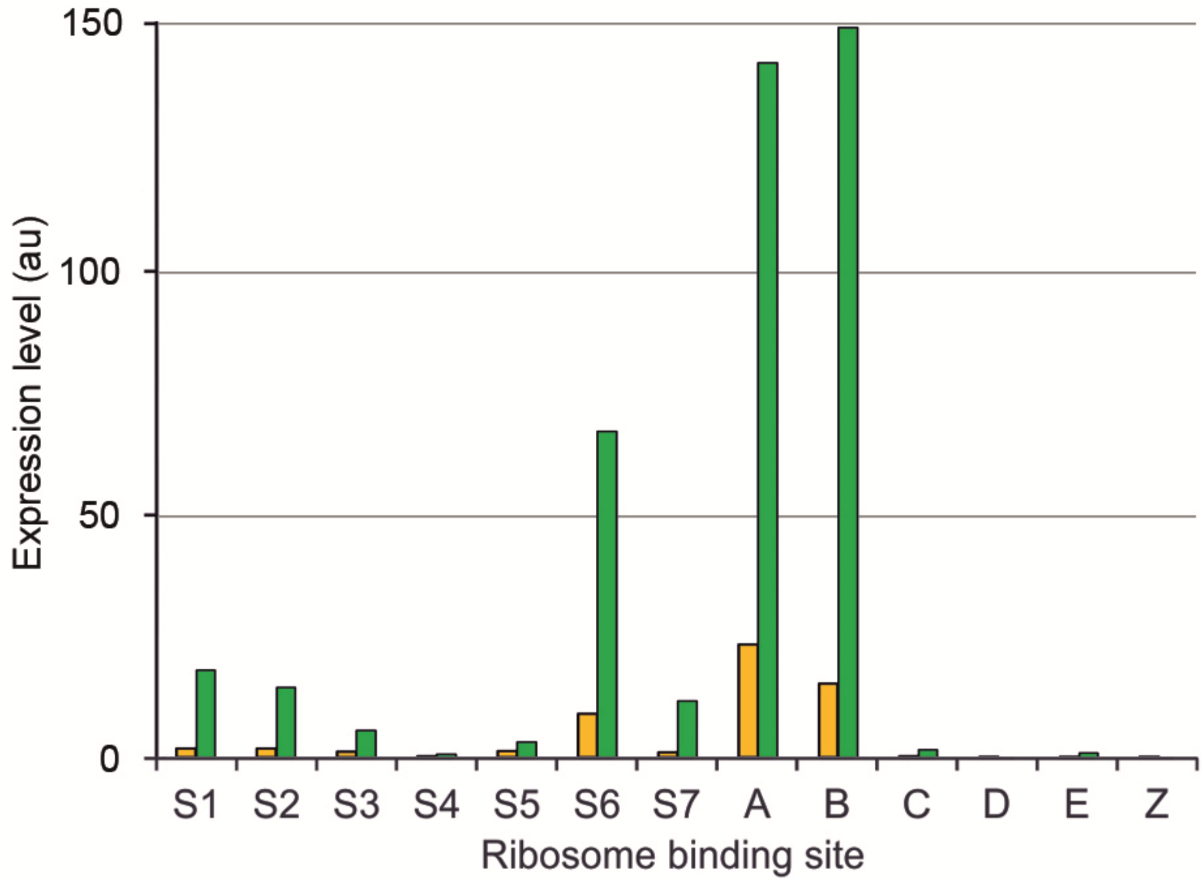

Additional file 7: Expression levels predicted by UTR Designer for sYFP2 (yellow) and GFPmut3b (green) in combination with the 13 target RBS sequences used in the study, with the nucleotide sequence around the start codon (-25 to +35) used as input.
